# Supplementary material for: High-altitude hypoxia exposure inhibits erythrophagocytosis by inducing macrophage ferroptosis in the spleen
Source: eLife. 2024 Apr 17;12:RP87496. doi: 10.7554/eLife.87496 (PMC11023697; doi:10.7554/eLife.87496)

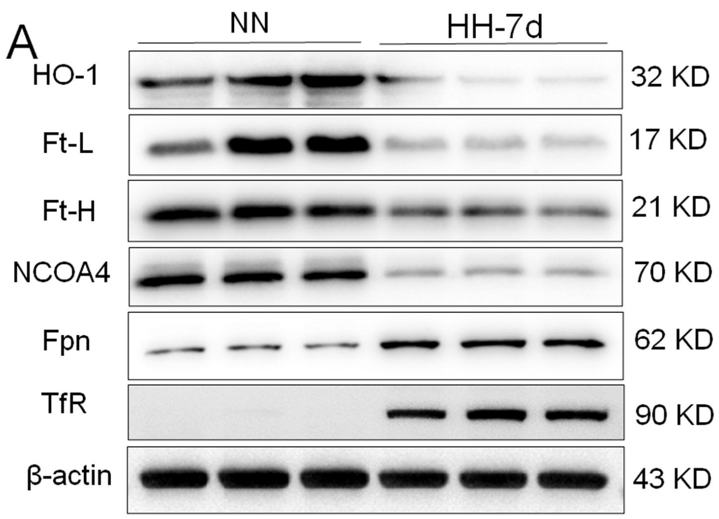

Figure 8A-7d-HO-1-32KD

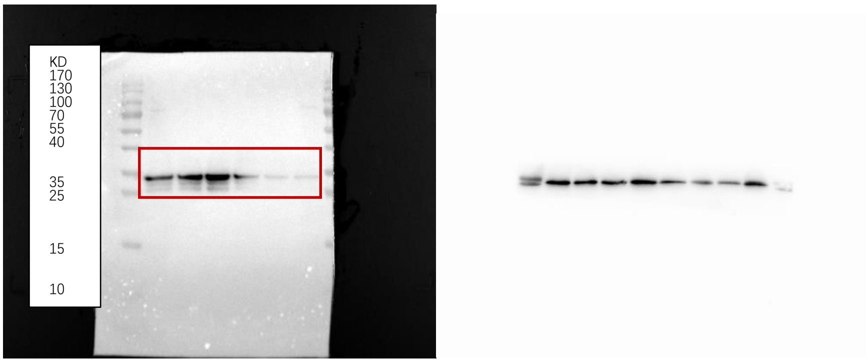

Figure 8A-7d-TfR-90KD

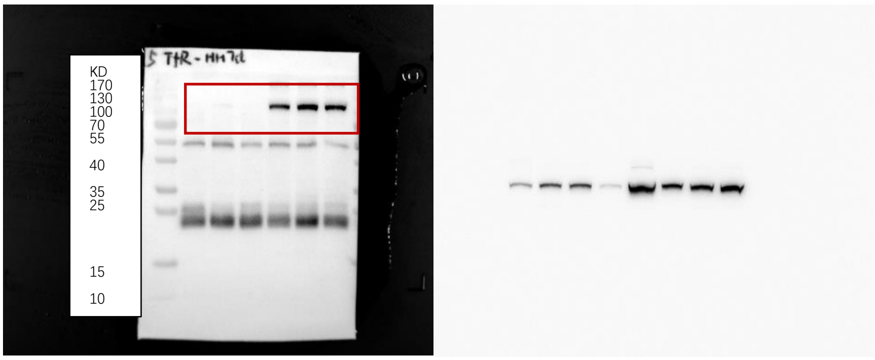

Figure 8A-7d-Ft-H-21KD

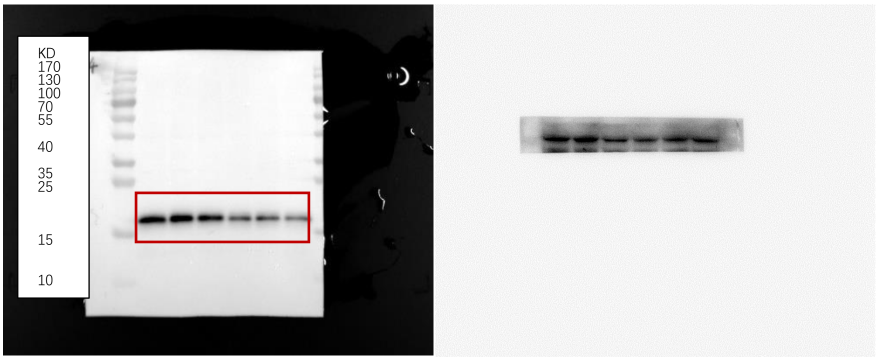

Figure 8A-7d-Ft-L-17KD

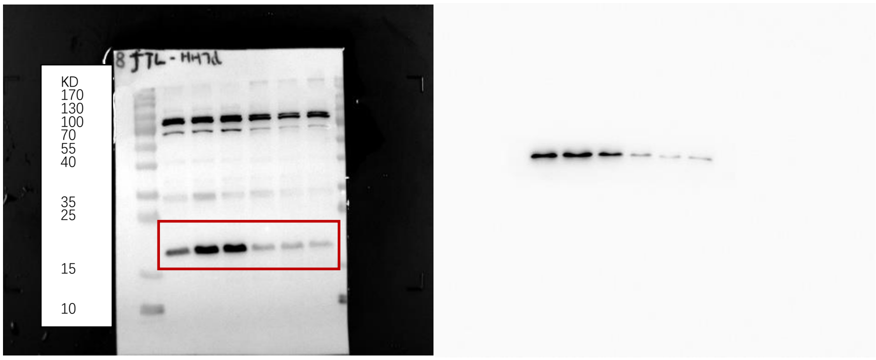

Figure 8A-7d-Fpn-62KD

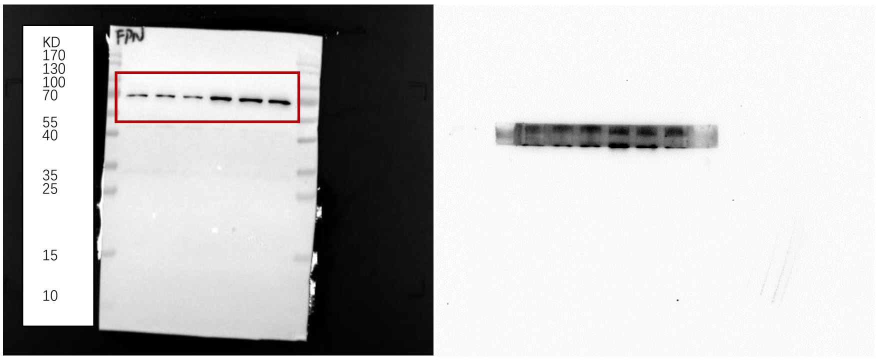

Figure 8A-7d-NCOA4-70KD

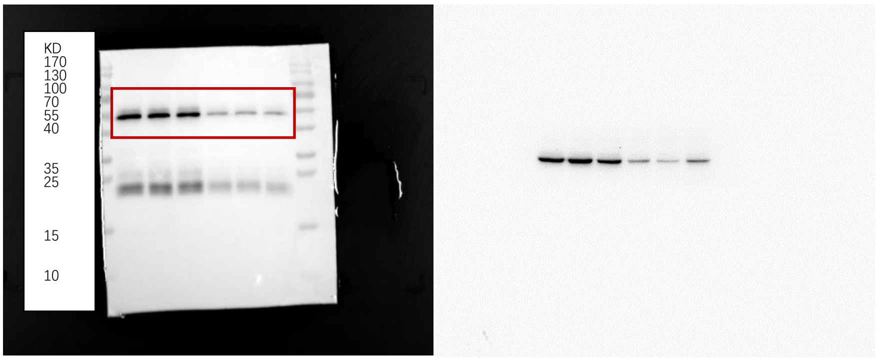

Figure 8A-7d-β-actin-43KD

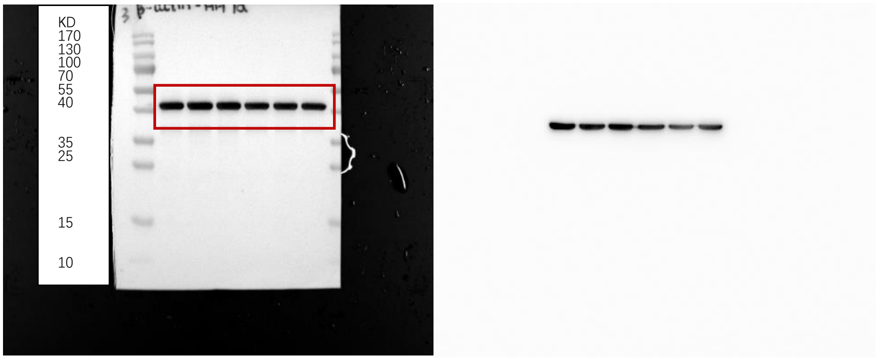

Supplement: Figure 8—source data 1. [file elife-87496-fig8-data1.zip › Figure 8-Source Data 1/Figure 8A-Source Data 1.pdf]
