# Supplementary figures and images for: High-altitude hypoxia exposure inhibits erythrophagocytosis by inducing macrophage ferroptosis in the spleen
Source: eLife. 2024 Apr 17;12:RP87496. doi: 10.7554/eLife.87496 (PMC11023697; doi:10.7554/eLife.87496)

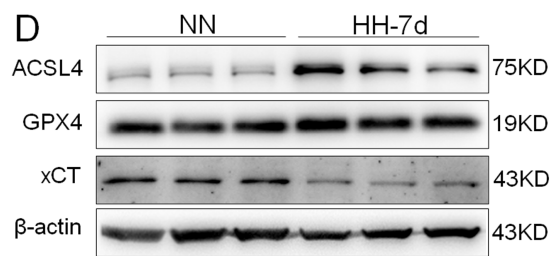

Figure 8D-7d-ACSL4-75KD

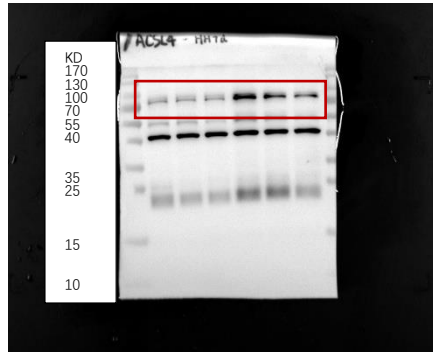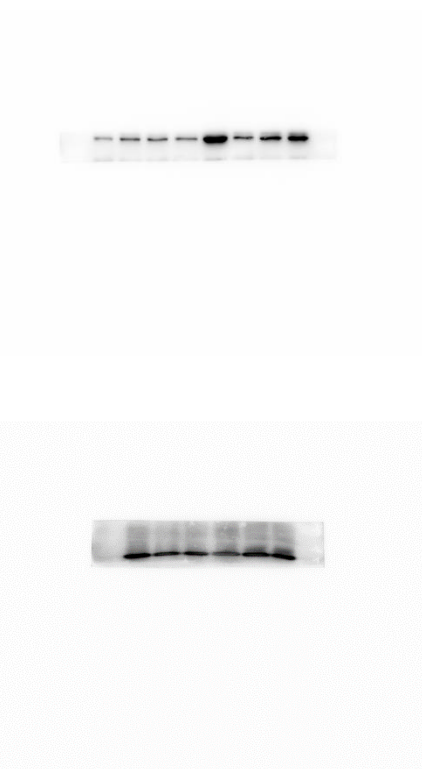

Figure 8D-7d-GPX4-19KD

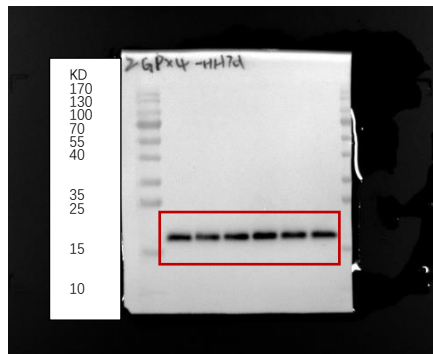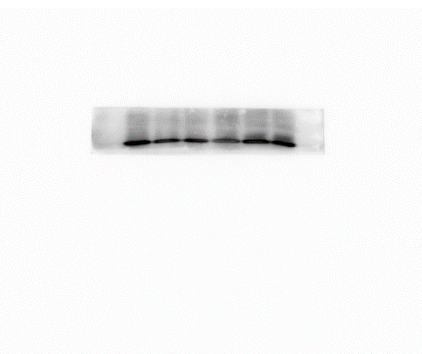

Figure 8D-7d-xCT -43KD

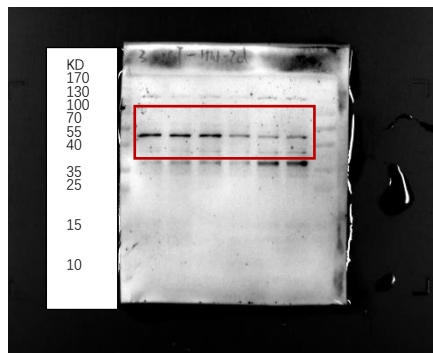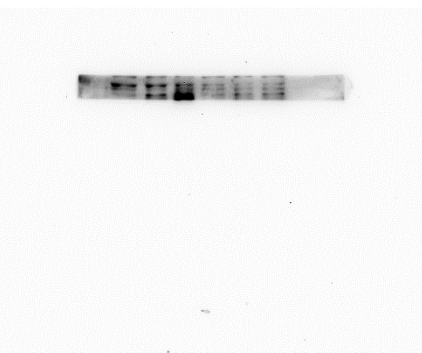

Figure 8D-7d-actin-43KD

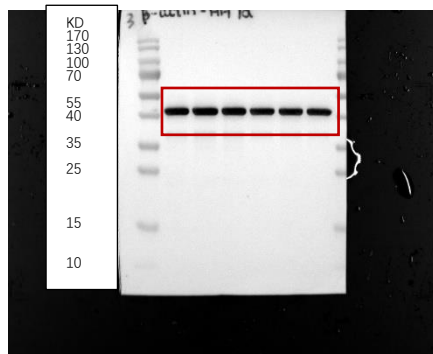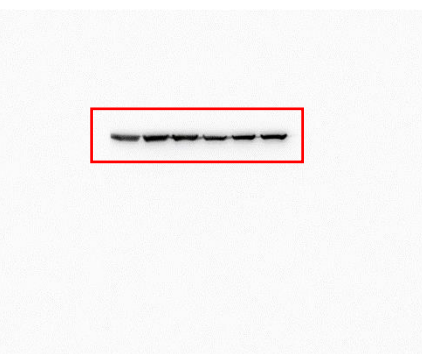

Supplement: Figure 8—source data 2. [file elife-87496-fig8-data2.zip › Figure 8-Source Data 2/Figure 8D-Source Data 2.pdf]

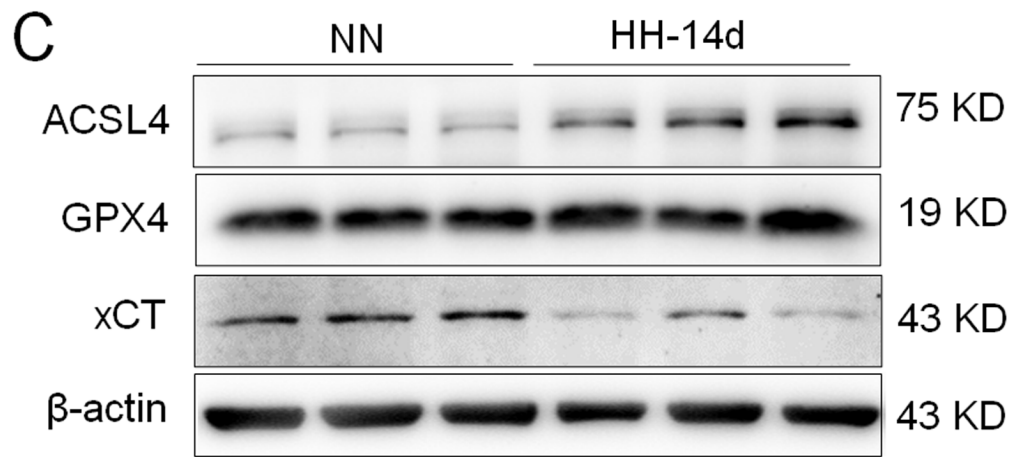

Figure S1C-14d-ACSL4

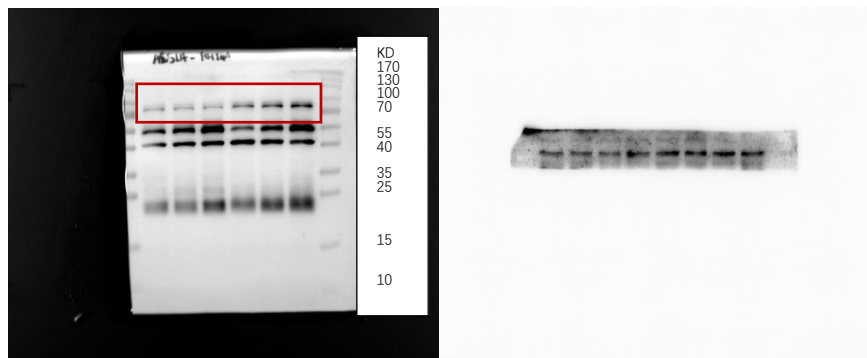

Figure S1C-14d-GPX4-19KD

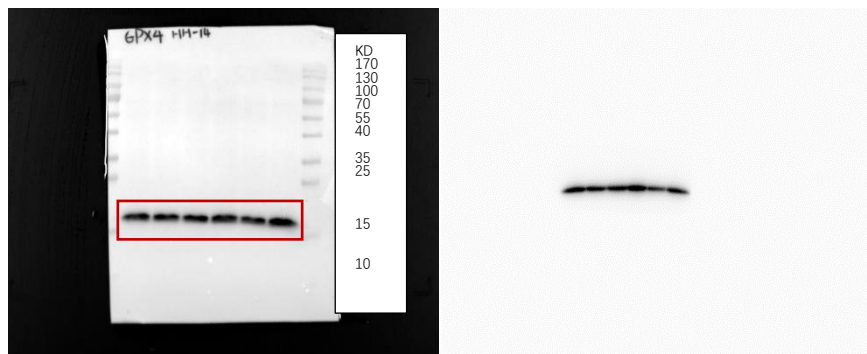

Figure S1C-14d-xCT-43KD

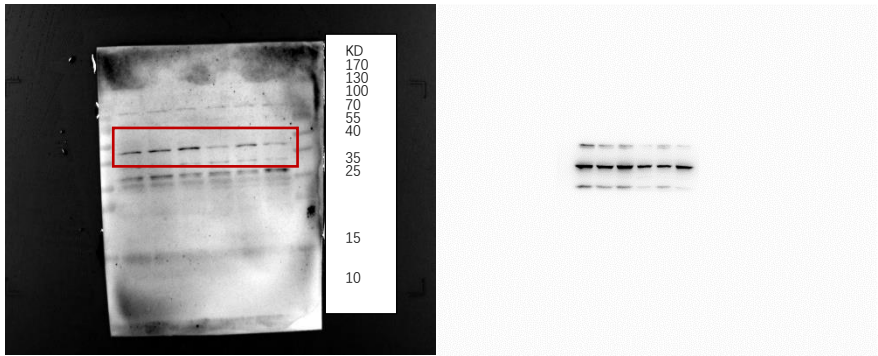

Figure S1C-14d-β-actin

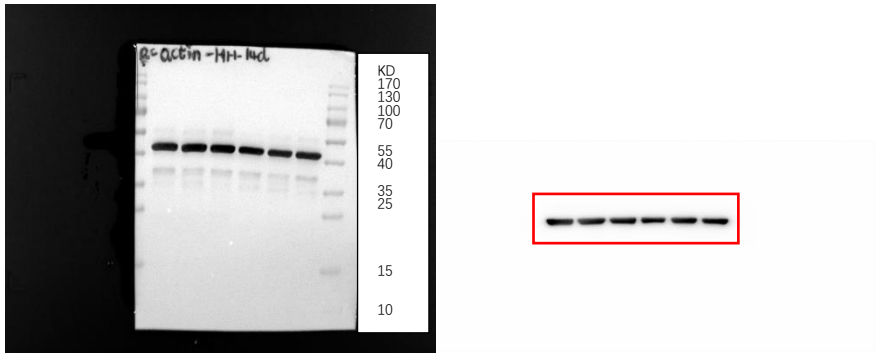

Supplement: Figure 8—figure supplement 1—source data 2. [file elife-87496-fig8-figsupp1-data2.zip › Figure 8-Figure supplement 1-Source data 2/Figure 8-Figure supplement 1C-Source data 2.pdf]

Figure 9F-ACSL4-75KD

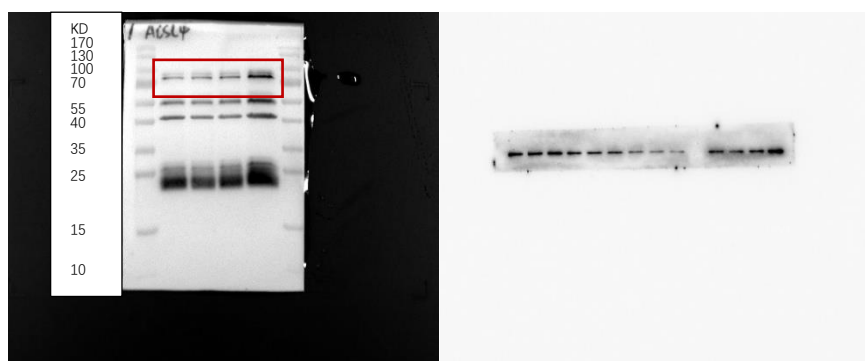

Figure 9F-xCT-43KD

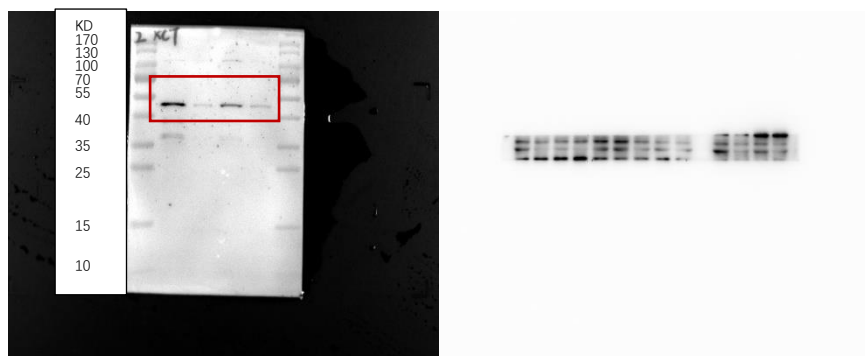

Figure 9F-GPX4-19KD

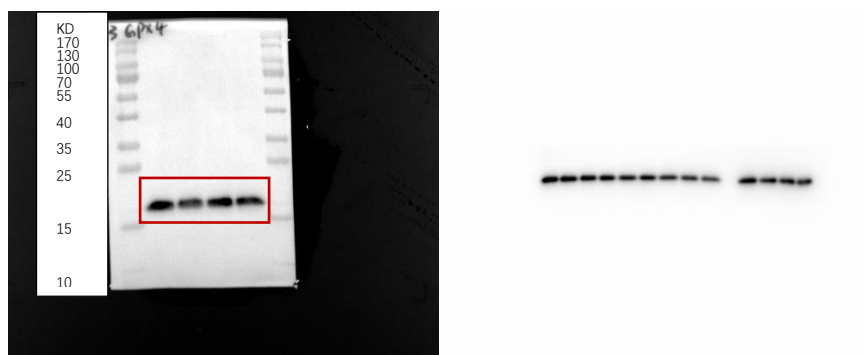

Figure 9F-actin-43KD

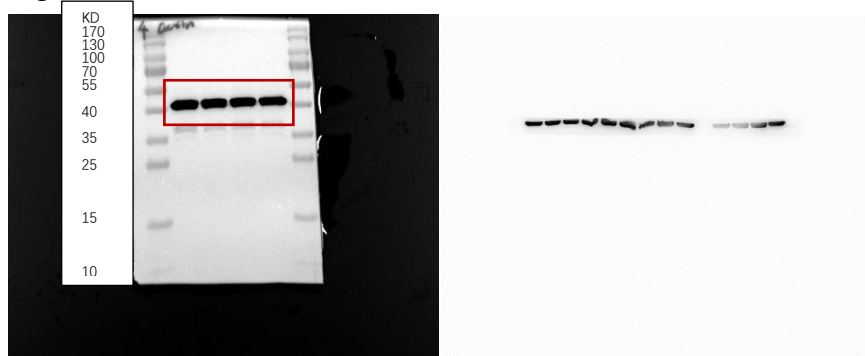

**F**

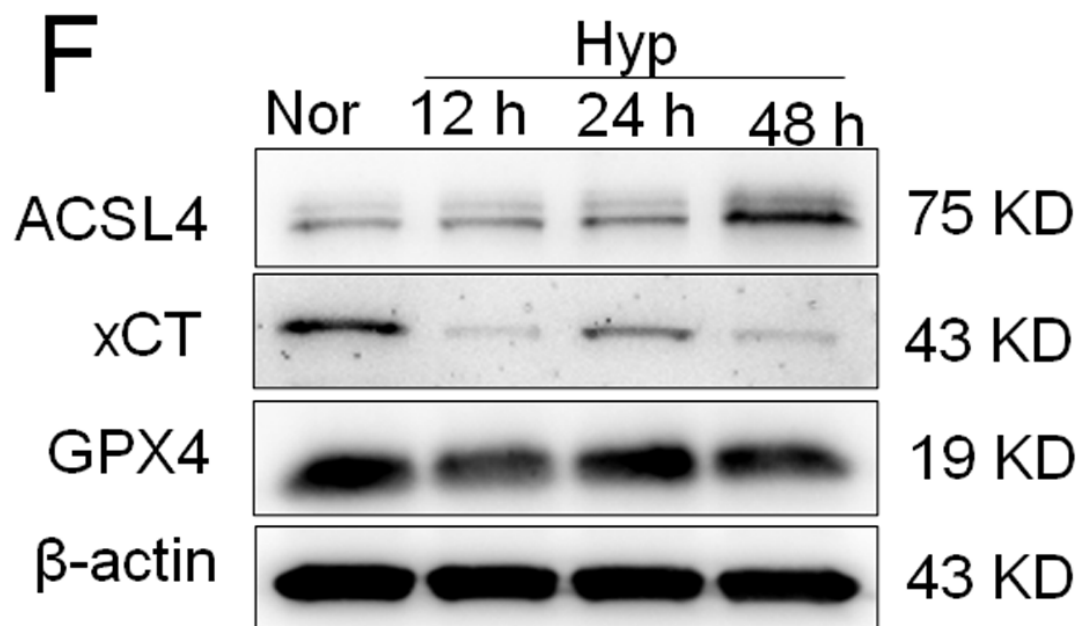

Supplement: Figure 9—source data 1. [file elife-87496-fig9-data1.zip › Figure 9-Source Data 1/Figure 9F.pdf]

Figure 9L-ACSL4-75KD

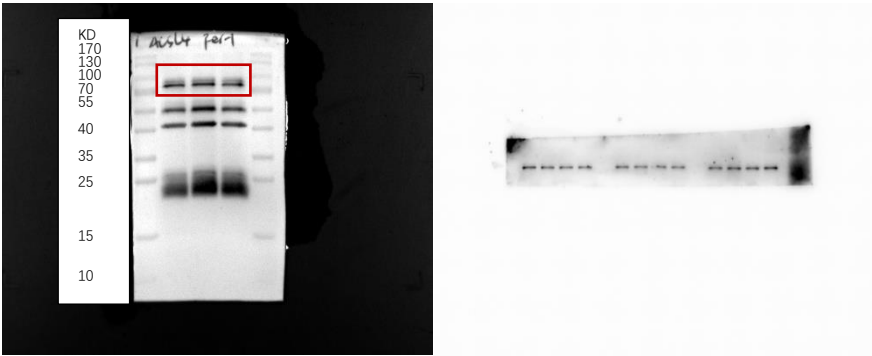

Figure 9L-xCT-43KD

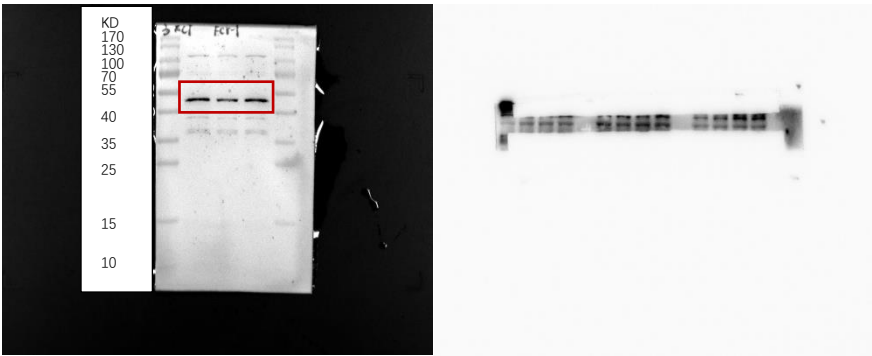

Figure 9L-GPX4-19KD

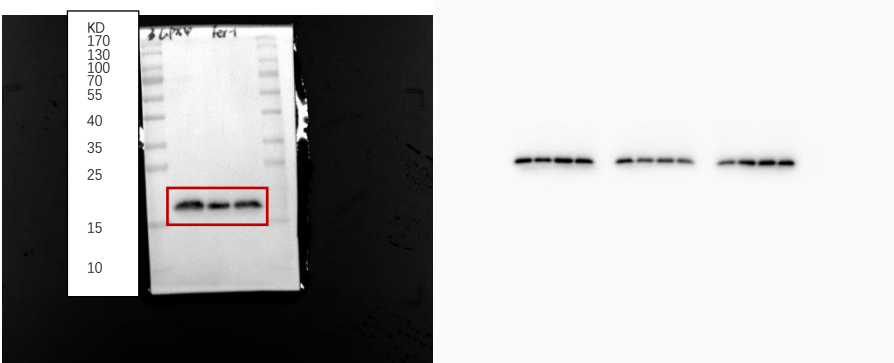

Figure 9L-actin-43KD

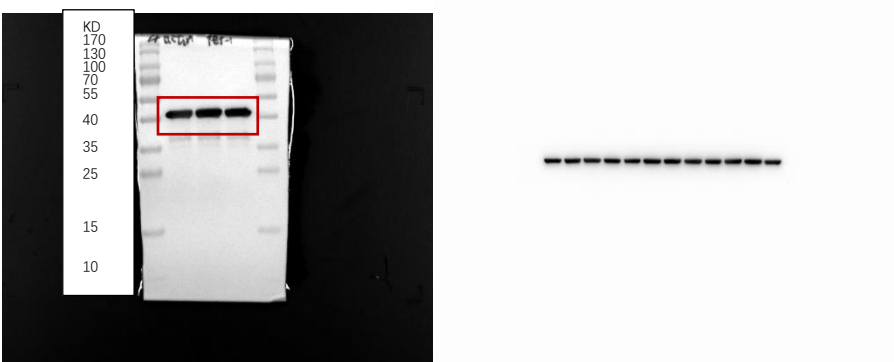

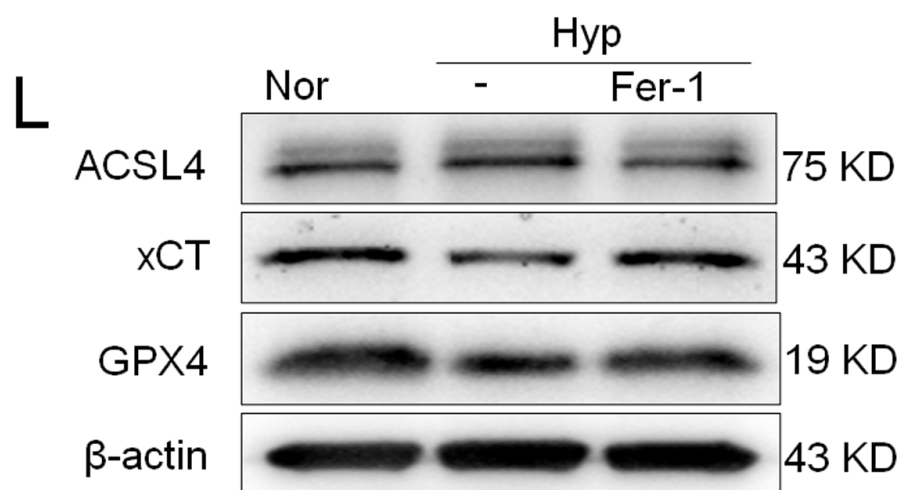

Supplement: Figure 9—source data 2. [file elife-87496-fig9-data2.zip › Figure 9-Source Data 2/Figure 9L.pdf]
