# Supplementary material for: High-altitude hypoxia exposure inhibits erythrophagocytosis by inducing macrophage ferroptosis in the spleen
Source: eLife. 2024 Apr 17;12:RP87496. doi: 10.7554/eLife.87496 (PMC11023697; doi:10.7554/eLife.87496)

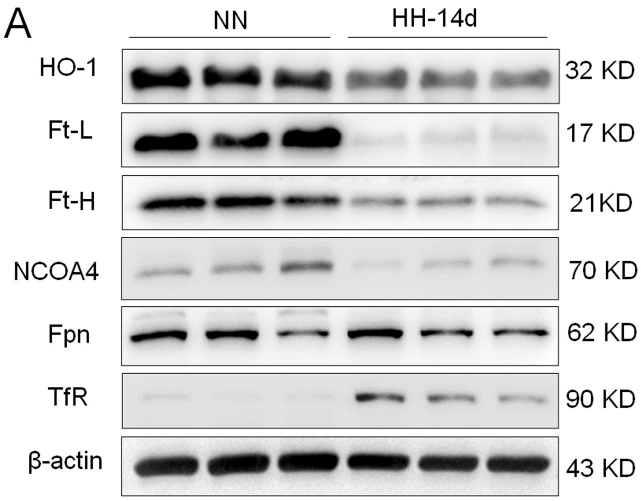

Figure S1A-14d-HO-1-32KD

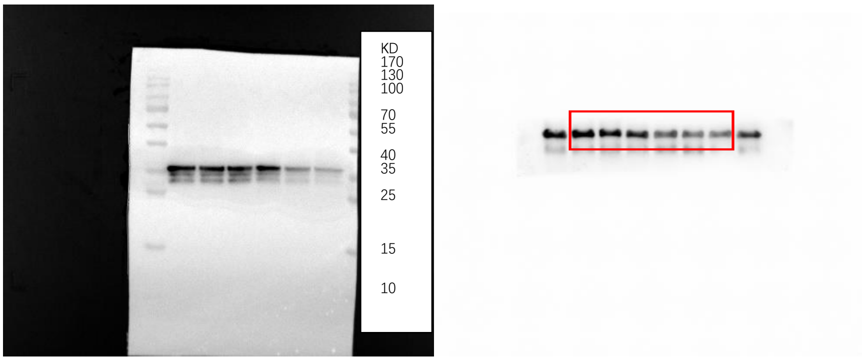

Figure S1A-14d-TfR-90KD

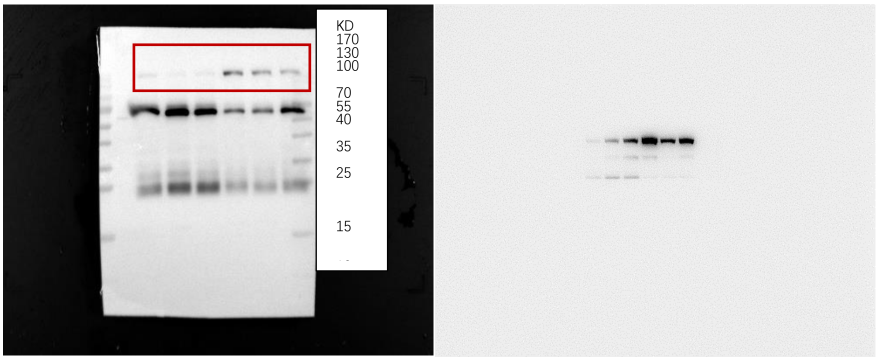

Figure S1A-14d-Ft-H-21KD

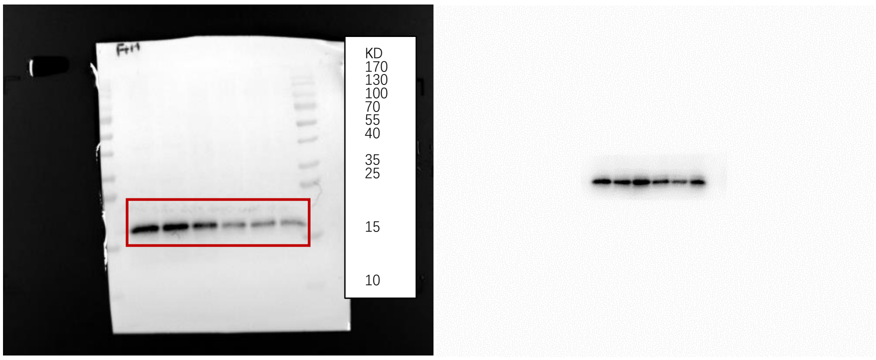

Figure S1A-14d-Ft-L-17KD

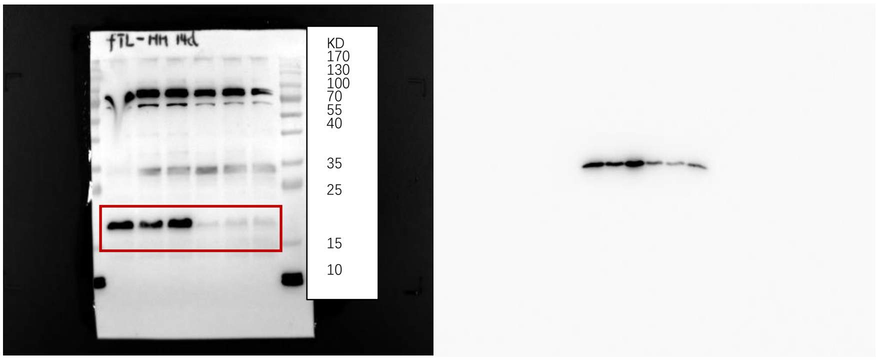

Figure S3A-14d-Fpn-62KD

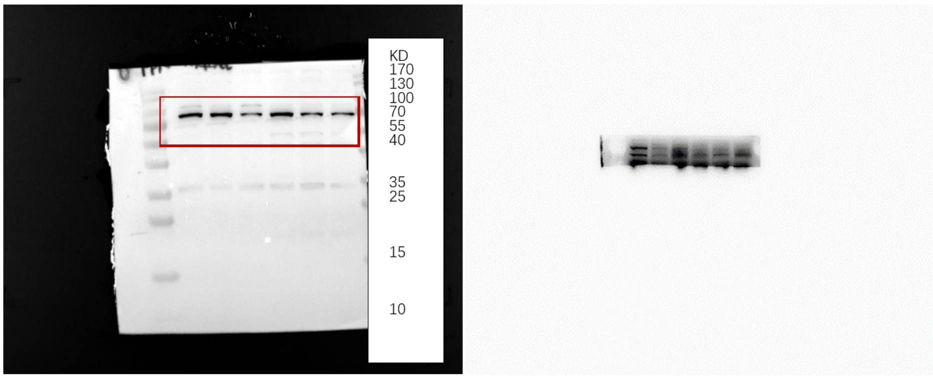

Figure S1A-14d-NCOA4-70KD

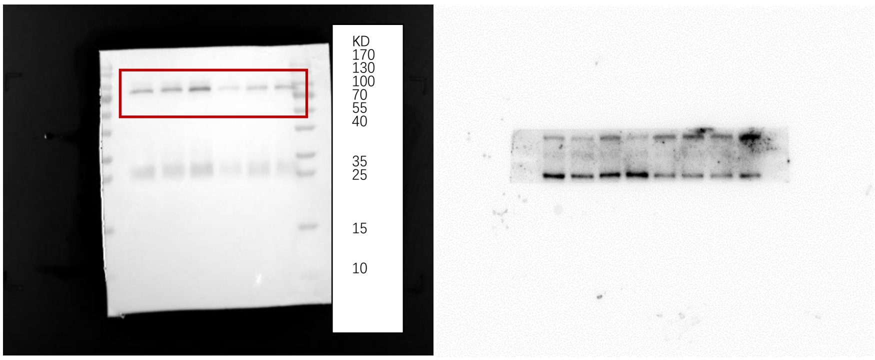

Figure S1-14d-β-actin-43KD

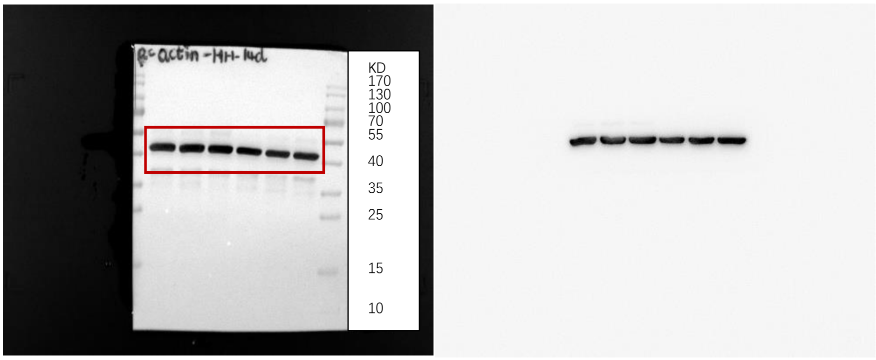

Supplement: Figure 8—figure supplement 1—source data 1. [file elife-87496-fig8-figsupp1-data1.zip › Figure 8-Figure supplement 1-Source data 1/Figure 8-Figure supplement 1A-Source data 1.pdf]
